# Fine-Tuning Methods for Large Language Models in Clinical Medicine by Supervised Fine-Tuning and Direct Preference Optimization: Comparative Evaluation

**DOI:** 10.2196/76048

**Published:** 2025-09-23

**Authors:** Thomas Savage, Stephen P Ma, Abdessalem Boukil, Ekanath Rangan, Vishwesh Patel, Ivan Lopez, Jonathan Chen

**Affiliations:** 1 Division of Hospital Medicine, Perelman School of Medicine, Department of Medicine, University of Pennsylvania, 3400 Spruce St, Philadelphia, PA, 19147, United States, 1 2155191670; 2 Division of Hospital Medicine, Department of Medicine, Stanford Medicine, Palo Alto, CA, United States; 3 Linguamind AI, Sousse, Tunisia; 4 Department of Medicine, Stanford Medicine, Palo Alto, CA, United States; 5 Department of Medicine, Saint Michael’s Medical Center, Newark, New Jersey, United States; 6 Center for Biomedical Informatics Research, Stanford University, Palo Alto, CA, United States; 7 Stanford Center for Biomedical Informatics Research, Palo Alto, CA, United States; 8 Clinical Excellence Research Center, Stanford University, Palo Alto, CA, United States

**Keywords:** artificial intelligence, direct preference optimization, supervised fine-tuning, fine-tuning, large language models

## Abstract

**Background:**

Large language model (LLM) fine-tuning is the process of adjusting out-of-the-box model weights using a dataset of interest. Fine-tuning can be a powerful technique to improve model performance in fields like medicine, where LLMs may have poor out-of-the-box performance. The 2 common fine-tuning techniques are supervised fine-tuning (SFT) and direct preference optimization (DPO); however, little guidance is available for when to apply either method within clinical medicine or health care operations.

**Objective:**

This study aims to investigate the benefits of fine-tuning with SFT and DPO across a range of core natural language tasks in medicine to better inform clinical informaticists when either technique should be deployed.

**Methods:**

We use Llama3 8B (Meta) and Mistral 7B v2 (Mistral AI) to compare the performance of SFT alone and DPO across 4 common natural language tasks in medicine. The tasks we evaluate include text classification, clinical reasoning, text summarization, and clinical triage.

**Results:**

Our results found clinical reasoning accuracy increased from 7% to 22% with base Llama3 and Mistral2, respectively, to 28% and 33% with SFT, and then 36% and 40% with DPO (*P*=.003 and *P*=.004, respectively). Summarization quality, graded on a 5-point Likert scale, was 4.11 with base Llama3 and 3.93 with base Mistral2. Performance increased to 4.21 and 3.98 with SFT and then 4.34 and 4.08 with DPO (*P*<.001). *F*_1_-scores for provider triage were 0.55 for Llama3 and 0.49 for Mistral2, which increased to 0.58 and 0.52 with SFT and 0.74 and 0.66 with DPO (*P*<.001). *F*_1_-scores for urgency triage were 0.81 for Llama3 and 0.88 for Mistral2, which decreased with SFT to 0.79 and 0.87, and then experienced mixed results with DPO, achieving 0.91 and 0.85, respectively (*P*<.001 and *P*>.99, respectively). Finally, *F*_1_-scores for text classification were 0.63 for Llama3 and 0.73 for Mistral2, which increased to 0.98 and 0.97 with SFT, and then essentially did not change with DPO to 0.95 and 0.97, respectively (*P*=.55 and *P*>.99, respectively). DPO fine-tuning required approximately 2 to 3 times more compute resources than SFT alone.

**Conclusions:**

SFT alone is sufficient for simple tasks such as rule-based text classification, while DPO after SFT improves performance on the more complex tasks of triage, clinical reasoning, and summarization. We postulate that SFT alone is sufficient for simple tasks because SFT strengthens simple word-association reasoning, whereas DPO enables deeper comprehension because it is trained with both positive and negative examples, enabling the model to recognize more complex patterns. Ultimately, our results help inform clinical informaticists when to deploy either fine-tuning method and encourage commercial LLM providers to offer DPO fine-tuning for commonly used proprietary LLMs in medicine.

## Introduction

### Overview

Large language models (LLMs) have sparked considerable interest in the medical field, offering potential for transformative clinical and operational applications [1-3]. However, to be effectively deployed in health care settings, these models often require additional refinement. While prompt engineering is a commonly used strategy for tailoring model behavior [4], it is not sufficient for all tasks. In cases where prompt engineering falls short, fine-tuning provides a more robust approach to adapt LLMs to specific medical use cases.

Fine-tuning is the process of adjusting the coefficient weights of a language model after pretraining, adapting the model with a subject-specific dataset of interest to the user [5-8]. To date, few LLM applications in medicine have deployed fine-tuning. In turn, there is a scarcity of literature informing users about which natural language processing (NLP) tasks benefit from LLM fine-tuning and, for those that benefit, which specific fine-tuning methods should be deployed. Therefore, in this study, we quantify the benefits of 2 common fine-tuning techniques, supervised fine tuning (SFT) and direct preference optimization (DPO), across a few key elementary tasks in clinical NLP.

### Background

SFT has been the conventional method of fine-tuning a language model. SFT requires the user to provide example prompts and desirable reference responses. SFT uses a classic loss function to adjust model weights and maximize the probability that the model will reproduce similar gold standard responses [9]. In many ways, SFT is simply training the model to mimic reference responses.

DPO is a variation of reinforcement learning that has become a popular fine-tuning technique because of its stability when training with smaller datasets [10]. In contrast to SFT, DPO requires the user to provide not only prompts and gold standard responses but also “rejected” (meaning less preferred) responses that the user finds undesirable. The use of rejected responses for fine-tuning is the key difference between SFT and DPO because DPO adjusts model weights to both maximize the likelihood of desired responses and minimize the likelihood of less preferred “rejected” responses. This conceptual difference is reflected in the DPO loss function (Multimedia Appendix 1) [5,9,10]. DPO is typically used on a model that has already undergone SFT fine-tuning.

When to use DPO is an area of active investigation. DPO is described as providing better alignment with human preferences, but recent publications have highlighted the ambiguity of this description [9]. It is unknown whether better alignment translates to better reasoning, summarization, information retrieval, or other tasks of importance to clinicians. Overall, few studies have compared SFT with DPO for individual NLP tasks important to medicine [5].

To address these gaps, our study aims to test key clinical NLP tasks for benefit from SFT and DPO fine-tuning. Specifically, we evaluate simple classification, clinical reasoning, text summarization, and clinical triage—areas where enhanced language model capabilities could meaningfully support medical decision-making.

## Methods

### Overview

We compared SFT and DPO on 4 datasets, each evaluating a core clinical NLP task. A glossary of terms is provided in Multimedia Appendix 2. We performed our investigation on 2 popular open-source LLMs, Llama3-8B-Instruct [11] and Mistral-Instruct-v2 [12], using datasets of fewer than 5000 training examples.

**Figure 1.**
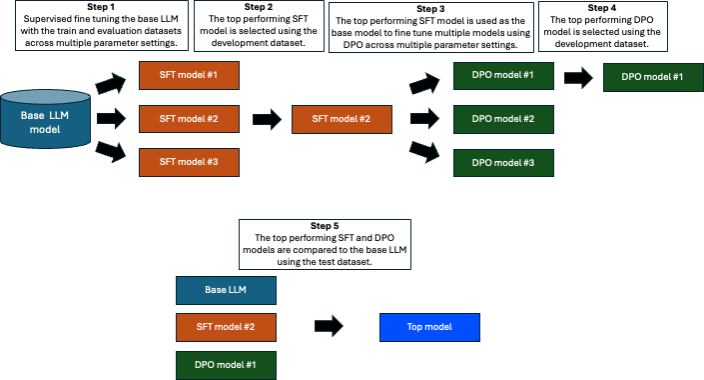
Overview of the methods used to fine-tune the SFT and DPO models, as well as compare the fine-tuned models with the base large language model. DPO: direct preference optimization; LLM: large language model; SFT: supervised fine-tuning.

Each dataset consisted of a training, evaluation, development, and test set. The base LLM model was first fine-tuned via SFT using the training and evaluation datasets, and then the development dataset was used to select the top-performing SFT model. The top-performing SFT model was then used as the base model for DPO fine-tuning. DPO was then performed using the train and evaluation datasets, and the top-performing DPO model was selected using the development set. Finally, the base LLM, top-performing SFT model, and top-performing DPO model were compared using the test set. This evaluation process is illustrated in Figure 1.

### Elementary Tasks Evaluated

The 4 elemental NLP tasks of interest were selected for evaluation from the systematic review by Bedi et al [2]: simple classification, clinical reasoning, text summarization, and triage. Bedi et al [2] completed a review of 519 studies that used LLMs for medical applications and grouped them by overall task to identify how LLMs are used in clinical practice. From that list of tasks compiled by Bedi, we selected the tasks most likely to benefit from fine-tuning for inclusion in our study.

These 4 tasks reflect key functions that clinicians frequently perform in real-world settings. Simple classification is used to categorize clinical notes for purposes such as billing, quality reporting, or operational workflows [1,13]. Clinical reasoning tasks require the model to interpret clinical information—such as patient histories or provider notes—and generate diagnostic assessments or treatment recommendations [14-16]. Summarization helps clinicians condense lengthy documentation into concise, high-yield summaries to support faster chart review [17]. Finally, triage tasks apply abstract, nonexplicit criteria to determine case prioritization, such as identifying patients who need urgent evaluation or allocating limited resources in emergency or ambulatory care settings [18].

Below we describe our methods used to evaluate each task. Table 1 provides additional details on the dataset used for each task.

**Table 1.** Description of the NLP tasks evaluated and the corresponding dataset, gold standard answer, and rejected answer. The same datasets and preferred samples were used for both SFT and DPO. All datasets (except for patient message triage) are provided in MultimediaAppendices 3^7^.

Tasks	Description	Clinical scenario tested	Dataset	Preferred sample	Rejected sample
Simple classification	Recognize a strict text-based criterion to classify a passage into one of multiple groups.	Identify passages describing patients with a UTI^a^ (pyuria with lower urinary tract symptoms) versus only pyuria.	Total dataset size: 700 patient scenarios were generated by GPT-4 [19] and then edited by 3 board-certified physicians for accuracy and to provide sufficient data variability.	Diagnosis by a board-certified physician.	Incorrect diagnosis not selected by grading physician.
Clinical triage	Recognize an abstract criterion to classify a passage into one of multiple groups.	Triage patient messages for both the appropriate urgency of response (urgent or nonurgent) and appropriate responding provider (physician or medical assistant).	Total dataset size: 1800 outpatient clinic patient messages from Stanford Health Care triaged by physician author TRS according to criteria listed in Multimedia Appendix 7.	Appropriate triage as determined by the grading physician (author TRS).	Incorrect triage not selected by the grading physician.
Clinical reasoning	Interpret patient information to identify diagnoses and select treatments.	Medical board exam questions evaluating the skills of clinical diagnosis and treatment selection.	Total dataset size: 5161 MedQA dataset [20], modified to questions evaluating clinical diagnosis and treatment selection at the step 2 and 3 level [21,22].	Correct answer provided by the MedQA dataset.	Randomly selected incorrect multiple-choice option provided by the MedQA dataset.
Summarization	Identify key information in a passage for a target audience.	Summarize a discharge summary note into 2‐3 sentences for an internal medicine physician.	Total dataset size: 5250 synthetic discharge notes from the AISC Augmented Clinical Notes dataset [23].	GPT-4 [19]–generated summaries.	Llama2 [24]–generated summaries.

a UTI: urinary tract infection

### Simple Classification

The first elementary task evaluated was simple classification, where we asked models to identify passages describing patients with a possible urinary tract infection (UTI). To be classified as a UTI, the passage needed to describe both pyuria and lower urinary tract symptoms [25,26].

The dataset was generated by GPT-4, which was prompted to generate 400 cases describing pyuria with no symptoms and 400 cases describing pyuria with urinary symptoms (positive for UTI). The 3 physician annotators then reviewed the generated cases to ensure correctness and introduce sufficient variability among the examples. The 800 examples were then split into a training set (300 examples), evaluation set (200 examples), development set (100 examples), and test set (200 examples). Prompts, patient descriptions, and model responses with grades are provided in Multimedia Appendix 3.

### Clinical Reasoning

The second elementary task evaluated was clinical reasoning. Clinical reasoning was evaluated using a modified MedQA dataset, where the original MedQA questions were adapted to be open-ended and included only step 2 and 3 level board exam questions (assessments that focus on higher levels of clinical reasoning).

The modified MedQA dataset consisted of 4095 training examples, 456 evaluation examples, 200 development examples, and 410 test questions. Reference answers were identified as the original MedQA answer, and rejected answers (used for DPO fine-tuning) were randomly selected from the list of incorrect multiple-choice options from the original dataset.

Each open-ended question was graded by at least 2 physician annotators. A question was marked correct if the answer provided was equivalent or equally correct to the gold standard answer provided by the MedQA answer key. If there was disagreement over the grade given by the first 2 physician annotators, the third annotator determined the final grade. The full data, along with the graded model responses, can be found in Multimedia Appendix 4.

### Summarization

The third elementary task evaluated was summarization, where the models were asked to summarize discharge summaries into 2‐3 sentences. Synthetic discharge summary notes were taken from the AISC Augmented Clinical Notes dataset [23]. Gold standard summaries were generated by GPT-4 (gpt-4‐0613) [19], and rejected examples for DPO fine-tuning were generated by the Llama2-chat-7B model Multimedia Appendix 5 [27]

The dataset consisted of 4,500 training examples, 300 evaluation examples, 150 development examples, and 300 test examples. LLM summaries were judged by GPT-4 (leveraging a state-of-the-art model as a judge is common practice within computer science [28-30]) on a five-point Likert scale, with 5 being the best possible score. The full data along with the model grades can be found in Multimedia Appendix 6.

### Triage

The final elementary task evaluated was triage, where the model was asked to triage patient messages for appropriate urgency (urgent vs nonurgent) and the appropriate responding provider (medical assistant vs physician). Patient messages were sourced from Stanford Clinics and graded by author TRS using the criteria provided in Multimedia Appendix 7.

A total of 2400 messages were graded. Messages that were ambiguous or did not require a response were not included in our investigation. The final dataset consisted of 1300 training examples, 200 evaluation examples, 100 development examples, and 200 test examples.

### Fine-Tuning Hyperparameters

Hyperparameters were tested with a sweep across a range, and the optimal settings were determined by testing on the development set. The learning rates tested were 10^−5^, 10^−6^, 10^−7^, and 10^−8^. The beta values tested were 0.1, 0.3, and 0.5.

Each model–hyperparameter configuration was initially tested with 1000 steps. The validation error plot was then analyzed to identify where the validation error plateaued, and the model was trained a second time with that step count.

All models produced by this investigation (with the exception of patient message triage) are available at the huggingface account *tsavage68*. Training was completed with the following python libraries: Transformers 4.44.2, Pytorch 2.4.0, Datasets 2.21.0, and Tokenizers 0.19.1.

### Statistical Evaluation

McNemar test was used for the statistical evaluation of tasks with binary outcomes (classification with text data, clinical reasoning, and triage). A 2-tailed paired t test was used for the statistical evaluation of tasks with ordinal outcomes (summarization). An α of .05 was used as our statistical significance threshold; however, accounting for 5 total tasks by the Bonferroni correction [31], we used a *P* value threshold of .01.

### Ethical Considerations

Patient messages were sourced from Stanford Health Care outpatient clinics under Stanford University Institutional Review Board Protocols 47618 and 76483, which approved the use of these data for research and quality improvement purposes. All data were deidentified to ensure patient confidentiality. Investigations with patient message data were performed on a Health Insurance Portability and Accountability Act–secure Google Cloud Platform account through Stanford University, and resulting models are not shared publicly.

## Results

### Simple Classification

**Figure 2.**
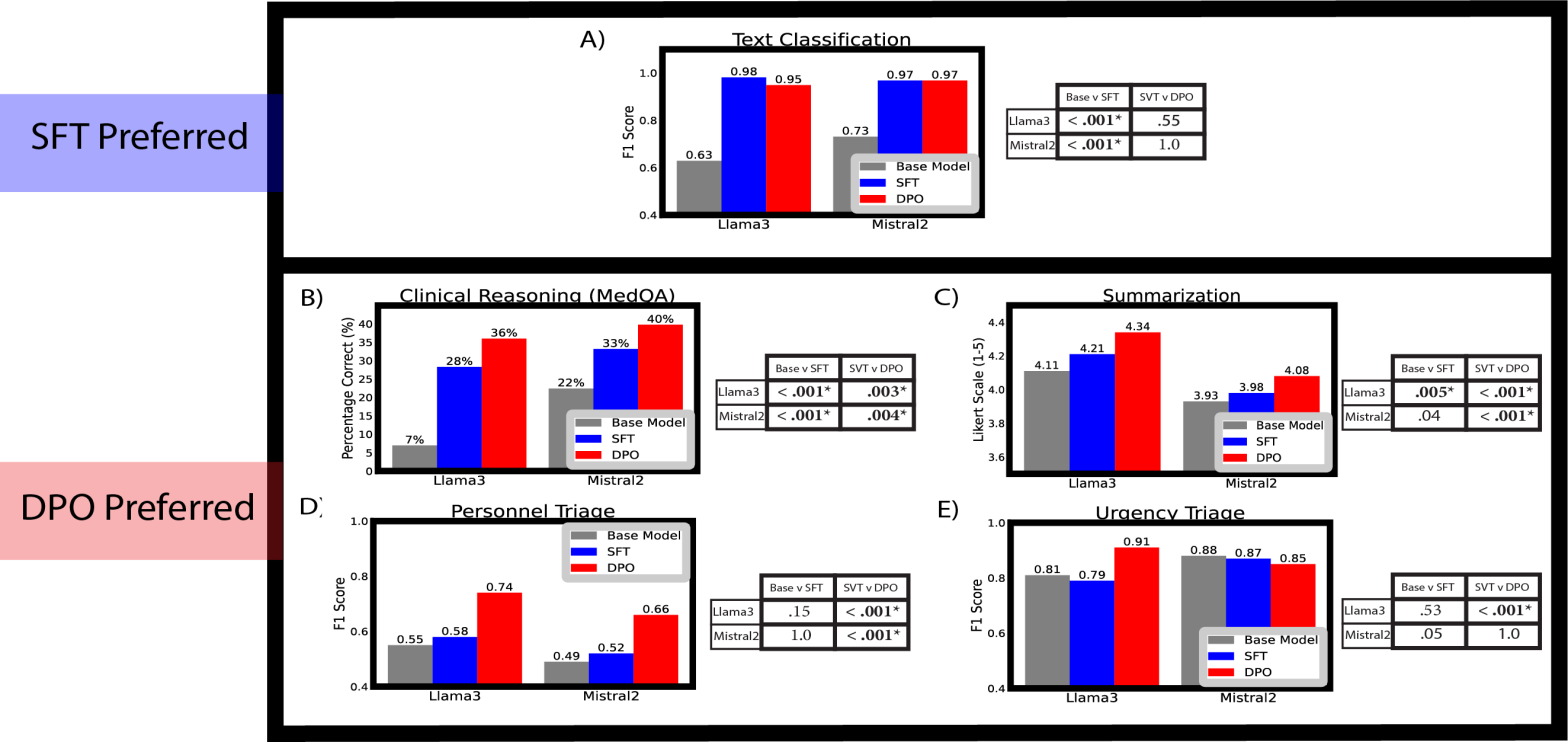
Comparison of base Llama3 and Mistral2 (gray) against SFT (blue) and DPO (red) fine-tuned variants for the tasks of (A) simple classification, (B) clinical reasoning, (C) summarization, and (D-E) triage. *P* values comparing model variants are provided to the right of each bar graph. Statistically significant *P* values are bolded with an asterisk. A *P* value of .01 was used to account for 5 total tasks by the Bonferroni correction. A definition of *F*_1_-score is provided in our glossary of terms. DPO: direct preference optimization; SFT: supervised fine-tuning.

In the classification with text data task, we found base Llama3 and Mistral2 achieved *F*_1_-scores of 0.63 and 0.73, respectively, when identifying passages describing patients with a UTI. With SFT, Llama3’s *F*_1_-score increased to 0.98 (*P*<.001), whereas Mistral2 increased to 0.97 (*P*<.001). With DPO fine-tuning, Llama3’s *F*_1_-score decreased to 0.95 (*P*=.55 compared to SFT), and Mistral2’s *F*_1_-score remained 0.97 (*P>*.99 compared to SFT). Results are provided in Figure 2A.

### Clinical Reasoning

In the clinical reasoning task, Llama3 and Mistral achieved accuracies of 7% and 22% respectively on a modified MedQA dataset. With SFT, the model accuracies increased to 28% (*P*<.001) and 33% (*P*<.001), respectively. With DPO, the model accuracies increased even further to 36% (*P*=.003) for Llama3 and 40% (*P*=.004) for Mistral2. The results are illustrated in Figure 2B. There was 97.2% agreement between the 2 grading physicians, and a third tie-breaking physician was only needed in 2.8% of questions.

### Clinical Summarization

In the clinical summarization task, Llama3 achieved an average five-point Likert scale rating of 4.11, and Mistral achieved a rating of 3.93, with 5 being the highest score and one the lowest. With SFT, ratings improved to 4.21 (*P*=.005) for Llama3 and 3.98 (*P*=.04) for Mistral2. With DPO, ratings further improved to 4.34 (*P*<.001) for Llama3 and 4.08 (*P*<.001) for Mistral2. The results are shown in Figure 2C.

### Clinical Triage

In the triage task, we found base Llama3 achieved *F*_1_-scores of 0.55 and 0.81 for personnel and urgency triage, respectively, whereas base Mistral2 achieved *F*_1_-scores of 0.49 and 0.88. With SFT, Llama3’s *F*_1_-score increased to 0.58 (*P*=.15) for personnel triage, but its *F*_1_-score decreased for urgency triage to 0.79 (*P*=.53). With SFT, Mistral2’s personnel triage *F*_1_-score increased to 0.58 (*P*>.99), and the urgency triage *F*_1_-score decreased to 0.87 (*P*=.05). With DPO, Llama3’s personnel triage *F*_1_-score increased to 0.74 (*P*<.001), and the urgency triage *F*_1_-score increased to 0.91 (*P*<.001). With DPO, Mistral2’s personnel triage *F*_1_-score increased to 0.66 (*P*<.001), but its urgency triage *F*_1_-score did not benefit, decreasing to 0.85 (*P*>.99). Figure 2D and E show *F*_1_-score results. Sensitivity and specificity data are provided in Multimedia Appendix 8.

### Training Dynamics

Investigations were completed with a single A100 graphics processing unit. Across all tasks, DPO training required approximately 2 to 4 times as many graphics processing unit-hours as SFT. For example, completing 1000 training steps with SFT for text classification required approximately 20 minutes of computational time, while DPO required 50 minutes. Similarly, 1000 steps of text summarization training required approximately 50 minutes with SFT and 160 minutes with DPO.

## Discussion

### Principal Findings

The results of our investigation demonstrate how fine-tuning with SFT and DPO can improve performance on common clinical natural language tasks. We found that SFT alone was sufficient for text-based classification (Figure 2A), whereas performance on the more complex tasks of triage, clinical reasoning, and summarization significantly improved with DPO (Figure 2B, C, D, and E). This nuanced performance advantage with DPO after SFT is an important finding because as artificial intelligence workflows become more common in clinical practice, the use of DPO can translate to tangible benefits for patients and providers. Physicians may reduce their risks of diagnostic errors and find AI-generated summaries more useful, while patients could find their care more equitably and efficiently triaged and expedited.

We postulate that SFT alone is sufficient for simple classification but not for triage, clinical reasoning, or summarization because SFT strengthens simple “word-association” reasoning, whereas DPO enables more nuanced interpretation. Because SFT is trained on only desired reference responses, the model is conditioned to recognize high-yield words or basic concepts but not deeper comprehension. By comparison, DPO is trained with both positive and negative examples, and this contrast enables the model to recognize more complex patterns (mimicking better understanding). As a result, we observe that SFT alone is sufficient for classification tasks with clearly defined criteria, such as diagnosing a UTI, whereas DPO fine-tuning is better for classification tasks that have abstract criteria such as patient message triage, clinical reasoning, or summarization. It is important to note, however, that DPO requires approximately 2 to 4 times more computational resources than SFT alone. We conclude that while SFT is sufficient for simple tasks driven by word or entity association, DPO offers superior performance for tasks requiring recognition of more complex patterns—albeit at a higher computational cost.

### Future Directions

Despite its promise, broader adoption of DPO remains limited by the current software infrastructure. Most leading commercial LLM providers—including OpenAI, Google, and Anthropic—do not offer DPO fine-tuning as part of their platforms [32-34]. This lack of support restricts the ability to optimize high-performing models such as GPT-4 (OpenAI), Gemini (Google DeepMind), and Claude-3 (Anthropic) for clinical tasks where alignment with clinician expectations is critical. To unlock the full potential of LLMs in medicine, it is essential for the informatics community and technology providers to collaborate on developing tools and workflows that support DPO fine-tuning for real-world clinical applications.

### Limitations

One limitation of our investigation is the reliance on synthetic training data. While synthetic data enables sharing of results and models without the ethical risk of exposing protected health information or having to use patient personal data to develop an AI product without their consent, it introduces bias and lacks the full diversity present in real-world prospective clinical data. As such, we encourage future studies to validate our findings using real-world datasets to ensure generalizability to real-world clinical applications.

A second limitation of our investigation is that we did not evaluate language models with more than ten billion parameters, although the trend in our results is expected to be consistent, even for larger models. Our exploration of moderately sized models provides valuable insight to guide investment in fine-tuning larger models that will be used in clinical operations or care.

### Comparison to Prior Work

A notable strength of our investigation is the use of datasets with fewer than 5000 training examples to reflect the data limitations of clinical medicine. Many existing publications on fine-tuning deploy training sets of more than 30,000 examples [5,17,35,36], sizes that are unrealistic for a single hospital system or clinic to achieve. Therefore, our findings prove the feasibility of fine-tuning language models within the realistic data constraints of medicine.

### Conclusions

Fine-tuning with SFT alone is sufficient for simple classification tasks with well-defined criteria. In contrast, fine-tuning with DPO requires more computational resources, but better optimizes performance for complex tasks such as triage, clinical reasoning, and summarization.

## Supplementary material

10.2196/76048 Multimedia Appendix 1 Direct preference optimization loss function.

10.2196/76048 Multimedia Appendix 2 Glossary of terms.

10.2196/76048 Multimedia Appendix 3 Urinary tract infection classification files.

10.2196/76048 Multimedia Appendix 4 Clinical reasoning files.

10.2196/76048 Multimedia Appendix 5 Python code used to generate clinical summarization examples.

10.2196/76048 Multimedia Appendix 6 Summarization files.

10.2196/76048 Multimedia Appendix 7 Triage criteria.

10.2196/76048 Multimedia Appendix 8 Python code for supervised fine-tuning and direct preference optimization.

## Abbreviations

DPO: direct preference optimization
LLM: large language model
NLP: natural language processing
SFT: supervised fine-tuning
UTI: urinary tract infection
