# Supplementary material for: Fine-Tuning Methods for Large Language Models in Clinical Medicine by Supervised Fine-Tuning and Direct Preference Optimization: Comparative Evaluation
Source: J Med Internet Res. 2025 Sep 23;27:e76048. doi: 10.2196/76048 (PMC12457693; doi:10.2196/76048)
Supplement: Multimedia Appendix 1 [file jmir-v27-e76048-s001.docx]

This is the Direct preference optimization loss function. This function is the mathematical equation that DPO uses to train a model. The equation receives input “accepted” and “rejected” examples and calculates model updates so that the model produces outputs that are closer to the “accepted” examples and farther away from “rejected” examples.

$$\nabla_{\theta}L_{DPO}\left( \pi_{\theta}; \pi_{ref} \right)=$$

$$- \beta\mathbb{E}_{\left( x,y_{w},y_{l} \right)\sim D} \left[ \sigma\left( \hat{r}_{\theta}\left( x, y_{l} \right)- \hat{r}_{\theta}\left( x, y_{w} \right) \right) \left[ \nabla_{\theta} log\pi(y_{w} | x)- \nabla_{\theta} log\pi(y_{l} | x) \right] \right]$$

Key Variables Legend

**x**: The input prompt

**y_w_​**: The **preferred model output (“accepted” example).**

**y_l_​**: The less **preferred model output (“rejected” example).**

**D**: The dataset consisting of tuples

π_θ_​: The current model being fine tuned

π_ref_: The reference model

r_θ_: The reward function
