# Supplementary material for: Fine-Tuning Methods for Large Language Models in Clinical Medicine by Supervised Fine-Tuning and Direct Preference Optimization: Comparative Evaluation
Source: J Med Internet Res. 2025 Sep 23;27:e76048. doi: 10.2196/76048 (PMC12457693; doi:10.2196/76048)
Supplement: Multimedia Appendix 2 [file jmir-v27-e76048-s002.docx]

**Glossary of Terms**

Concepts included in our glossary of terms have a superscript g when listed in the text below.

**Hyperparameters**

A hyperparameter is a constant variable in the training process that controls an aspect of model training. The value of the hyperparameter is set by the user.

**Hyperparameter Sweep**

A hyperparameter sweep is the process of testing a range of hyperparameter values to identify the value that produces the best performing model.

**Loss Function**

**A loss function is a mathematical equation that quantifies how closely a model’s output matches a gold standard reference for a given input (prompt).** The resulting difference—known as the loss value—reflects the degree of difference between the predicted and desired outputs. This loss value guides how the model’s internal weights are updated, with the goal of producing outputs that more closely align with the gold standard.

**F1 Score**

An F1 score is a metric used in machine learning to evaluate the performance of a classification model. The equation for an F1 score is included below:

$$F1 Score= \frac{2*True Positives}{2*True Positives+False Positives+False Negatives}$$

**Validation Error Plateau**

In the process of fine tuning a large language model, the progressive model iterations are intermittently tested against a small validation test set. The maximal performance of the model is believed to have been achieved when the performance of model iterations on the validation set are no longer improving and have instead plateaued.

**Abbreviations**

DPO: Direct Preference Optimization

LLM: Large Language Model

NLP: Natural Language Processing

SFT: Supervised Fine Tuning
