# Supplementary material for: Fine-Tuning Methods for Large Language Models in Clinical Medicine by Supervised Fine-Tuning and Direct Preference Optimization: Comparative Evaluation
Source: J Med Internet Res. 2025 Sep 23;27:e76048. doi: 10.2196/76048 (PMC12457693; doi:10.2196/76048)
Supplement: Multimedia Appendix 5 [file jmir-v27-e76048-s005.docx]

import pandas as pd

# Load the Parquet file into a DataFrame

df = pd.read_parquet('train-00000-of-00001.parquet')

df_truncated = df.head(5500)

# Save the DataFrame to a CSV file

df_truncated.to_excel('aisc_augmented_synthetic_notes_first_5500.xlsx')

import pandas as pd

import numpy as np

# Seed set for reproducibility

np.random.seed(1)

# Shuffle the rows of the DataFrame

df_shuffled = df_truncated.sample(frac=1)

# Split the shuffled DataFrame into 4 separate DataFrames

train_df = df_shuffled.iloc[:4500]

evaluate_df = df_shuffled.iloc[4500:5000]

dev_df = df_shuffled.iloc[5000:5150]

test_df = df_shuffled.iloc[5150:5450]

# Save each DataFrame to an Excel file

train_df.to_excel('train_summary.xlsx', index=False)

evaluate_df.to_excel('evaluate_summary.xlsx', index=False)

dev_df.to_excel('dev_summary.xlsx', index=False)

test_df.to_excel('test_summary.xlsx', index=False)

eval_df=pd.read_excel('evaluate_summary.xlsx')

# Specify the column where you want to check the condition

column_name = 'rejected'

# Filter the DataFrame

filtered_df = train_df[~train_df[column_name].str.startswith(' The patient is a 74-year-old male with a history of')]

filtered_df.shape[0]

eval_df['chosen']= "nan"

eval_df['rejected']= "nan"

eval_df.reset_index(drop=True, inplace=True)

prompt1="""

Generate a clinical summary of the following note. The summary should be two sentences and include only pertinent information about the patient's past medical history, chief complaint and brief history of present illnesss. Only include information that a physician would find important.

---------------

"""

prompt2="""

Generate a clinical summary of the following note. The summary should be two sentences and include only pertinent information, first reporting the patient's demographics, then pertinent, past medical history, chief complaint/presentation, and then a brief history of present illnesss. Only include information that a physician would find important.

---------------

"""

prompt4="""

Generate a clinical summary of the following note. The summary should be two to three sentences and include only pertinent information, first reporting the patient's demographics, pertinent past medical problems or history (state if there is no pertinent past medical history), chief complaint/presentation, and then a brief history of present illnesss. Only include information that an internal medicine physician would find important.

---------------

"""

prompt3="""

Generate a problem list from the following note for an internal medicine physician. The problem list should include an explanation of every problem below its heading including a summary and assessment of each problem. Group and combine problems that are related. Only include information that an internal medicine physician would find important.

---------------

"""

prompt5="""

Generate a clinical summary of the following note. The summary should be two sentences and include only pertinent information, first reporting the patient's demographics, then pertinent past medical history, chief complaint/presentation, and then a brief history of present illnesss. Only include information that a physician would find important.

---------------

"""

prompt6="""

Generate a clinical summary of the following note. The summary should be three sentences and include only pertinent information, first reporting the patient's demographics, then pertinent past medical history, chief complaint/presentation, and then a brief history of present illnesss. Only include information that a physician would find important.

---------------

"""

#### THIS PROMPT IS THE ONE THAT WAS USED ######

prompt7="""

Generate a clinical summary of the following note. The summary should be three sentences and include only PERTINENT/Important information, first reporting the patient's demographics, then pertinent past medical history, chief complaint/presentation, and then a brief history of present illnesss. Only include information that a physician would find important.

---------------

"""

#### THIS EXAMPLE IS THE ONE THAT WAS USED ######

example_summary1 = """

Example Summary:

This is a 74 year old man with a past medical history of hypertension, chronic obstructive pulmonary disease, chronic kidney disease, coronary artery disease, and abdominal aortic aneurysm status post endovascular repair 8 years ago, admitted to the ICU for decompensated heart failure with pulmonary edema secondary to severe mitral regurgitation, treated with mitral valve repair. During the hospitalization a TEE was performed and complicated by hypopharyngeal injury without esophageal damage, resulting in a retropharyngeal abscess formation that responded to antibiotics and needle aspiration, the patient is currently tolerating a diet of small portions of soft food.

----------

Real Note

"""

example_summary2 = """

Example Problem List:

# Decompensated Heart Failure - Pulmonary Edema

# Mitral Regurgitation # Chordae rupture

Admission to the ICU for decompensated heart failure with pulmonary edema secondary to severe mitral regurgitation due to chordae rupture. the patient was treated with mitral valve repair with tricuspid annuloplasty with a St. jude Medical Tailor ring 31 mm.

# Hypopharyngeal Injury

# Right Parapharyngeal and Retropharyngeal absces

Hypopharyngeal injury secondary to injury during TEE procedure which resulted in formation of a parapharyngeal and retropharyngeal abscess. ENT nasolaryngoscopy showed no evidence of perforation or rupture in the hypopharyngeal cavity. The abscess was treated with piperacillin/tazobactam for 14 days and needle aspiration. The patient has been cleared to eat small portions of a soft diet.

# Atrial Fibrillation

# Coronary Artery Disease

# Chronic Obstructive Pulmonary Disease

# Chronic Kidney Disease

# Abdominal Aortic Aneurysm status post repair 8 years ago

----------

Real Note

"""

example_note = """

Example Note:

A 74-year-old man was referred to our cardiovascular center for the evaluation and treatment of mitral regurgitation (MR). He had been complaining of aggravation of dyspnea (New York Heart Association functional class IV) for the preceding 3 weeks, and atrial fibrillation and severe MR were detected from other clinic. The patient had medical history of hypertension and chronic obstructive pulmonary disease for 15 years, and received endovascular aneurysm repair for abdominal aortic aneurysm 8 years ago. He had coronary artery disease and chronic kidney disease, also.\nThe patient was admitted to the intensive care unit because of uncompensated heart failure and careful control of pulmonary edema with chronic kidney disease. We performed transthoracic echocardiography with subsequent TEE to comprehensive evaluation of mitral valve disease. The patient presented tachypnea and orthopnea before the TEE procedure, less than minimal dose of sedative agent was administrated to lessen patient's discomfort, 1 mg of lorazepam, 25 mg of fentanyl, intravenously. As we checked for the mental status, the patient was not sedate, before and during the insertion of TEE probe. The insertion of TEE probe was performed with the patient in left lateral decubitus position according to the following standardized technique: the probe was inserted through the midline and gently advanced to pass the first pharyngeal curvature corresponding to the base of the tongue. The probe was then extended, and the patient was asked to swallow, at which point the probe was further advanced to enter the esophageal inlet. When the probe has reached to the root of tongue, the patient suddenly changed his position form left decubitus to supine position and gave force to the neck, and resisted to probe insertion. The probe got lodged at right-side of hypopharyngeal area so that failed to advance. At second attempt, TEE probe was advanced into esophagus easily during swallowing with patient's cooperation. TEE demonstrated severe eccentric MR with medial commissural prolapse due to chordae rupture (A3-P3 commissure) with left ventricular dilatation, mild tricuspid regurgitation ().\nThe patient complained of painful throat and odynophagia after TEE. The physical examination revealed tenderness and crepitus on right anterolateral area of neck (Level III) 3 hours later after TEE. Subcutaneous emphysema was detected on subsequent neck X-ray (). Iatrogenic hypopharyngeal or esophageal injury was suspected, antibiotic treatment with piperacillin/tazobactam was initiated and the patient was not allowed to eat or drink. Computed tomography (CT) scan revealed subcutaneous emphysema without involvement of mediastinum, but the level of injury was not detected (). Esophagogram with gastrograffin swallowing showed no visible contrast leakage (). Fiberoptic nasolaryngoscopic examination identified edema of the right posterolateral wall of the hypopharynx and hypopharyngeal bruise but there was no evidence of rupture or perforation. Intravenous antibiotic therapy was continued and nasogastric tube feeding was done. Laboratory analysis revealed an elevation of the white blood cell count (20.36 × 103/µL) and C-reactive protein (CRP; 28.54 mg/dL).\nWe tried to find any evidence of esophageal injury because treatment strategy would be different if the esophagus was involved. An ENT specialist and a GI specialist agreed with hypopharyngeal injury without esophageal damage after multiple tests. We concluded the lesion was limited to the hypopharynx because the presence of hematoma at the right side of hypopharynx, consistent with the direction and the depth of the probe passage. Further, there was no evidence of esophageal injury on serial follow-up multimodality imaging studies.\nAfter 5 days from the injury, the subcutaneous emphysema disappeared and the patient remained afebrile with improvement of the leukocyte count and CRP (). However painful neck mass around anterolateral area of neck was noticed (Level III). The follow-up CT without contrast showed a right parapharyngeal and retropharyngeal abscess secondary to hypopharyngeal injury (). Ultrasound guided needle aspiration was done for abscess drainage. Yellowish fluid in the abscess was analyzed for cytology and revealed as acute inflammatory cells predominantly neutrophils. As the patient's systemic status was stable with decrease of the leukocyte count and CRP, the antibiotics (piperacillin/tazobactam) was considered to be susceptible. The antibiotic treatment with piperacillin/tazobactam was maintained for 14 days with the drainage of abscess because no organisms were identified from the abscess fluid culture and repeated blood cultures.\nOn 7th day after TEE, second swallowing study was performed and no leakage was demonstrated (), infectious parameters were continuously decreased, and follow-up fiberoptic nasolaryngoscopic examination demonstrated no evidence of perforation or rupture in hypopharyngeal cavity. So the patient was allowed to start eating small portions of soft food.\nAfter 14 days of antibiotic therapy, when the infection was controlled completely, the patient had successful mitral valve repair with tricuspid annuloplasty with St. Jude Medical Tailor ring 31 mm.

--------------

"""

!pip install openai

!pip install math

!pip install tiktoken

from openai import OpenAI

import os

client = OpenAI(api_key=os.environ.get("OPENAI_API_KEY", ***))

# Prompt A

for i in range(len(eval_df)):

print(i)

#########################################

# Prompt B

completion = client.chat.completions.create(

model="gpt-4o",

messages=[

{"role": "system", "content": "You are an expert physician."},

{"role": "user", "content": prompt6 + example_note + example_summary1 + eval_df['full_note'][i]}

],

logprobs=True,

#top_logprobs=1,

seed=1,

temperature = 0

)

eval_df['chosen'][i] = completion.choices[0].message.content

eval_df.to_csv('/content/drive/MyDrive/SFTvDPO/Summary_generation_good_eval2.csv', index=False)

from replicate.client import Client

replicate = Client(api_token=***)

train_df['rejected']= 'nan'

train_df = pd.read_csv('Summary_generation_good_eval1.csv')

from transformers import RobertaTokenizer

def truncate_to_tokens(text, max_tokens=4000):

# Initialize the tokenizer

tokenizer = RobertaTokenizer.from_pretrained('roberta-base')

# Tokenize the input text

tokens = tokenizer.tokenize(text)

# Check if the number of tokens exceeds the maximum limit

if len(tokens) > max_tokens:

# Truncate the tokens and re-convert to a string

truncated_tokens = tokens[:max_tokens]

truncated_text = tokenizer.convert_tokens_to_string(truncated_tokens)

return truncated_text

else:

# Return the original text if truncation is not necessary

return text

from os.path import expanduser

import time

for i in range(len(train_df)):

inter = train_df['rejected'][i]

substring = '74-year-old man'

if substring in inter:

prompt = train_df['full_note'][i]

print(i)

if len(prompt) > 15700:

prompt = prompt[:15700]

output = replicate.run(

"meta/llama-2-7b-chat",

input={

"debug": False,

"top_p": 1,

"prompt": prompt,

"temperature": 0.7,

"system_prompt": "You are an expert physician.",

"max_new_tokens": 512,

"min_new_tokens": -1

}

)

joined_string = "".join(output)

t = joined_string

train_df['rejected'][i] = t

train_df.to_csv('/content/drive/MyDrive/SFTvDPO/Summary_generation_bad_eval3.csv', index=False)

dev = pd.read_excel('dev_summary.xlsx')

#df = pd.read_excel('dev_aisc.xlsx')

dev['full_note'] = prompt6 + example_note + example_summary1 + dev['full_note'] + end

dev.to_excel('dev_summary_w_prompt.xlsx')

train_df['rejected'][0]

def is_nan_string(x):

return x == 'Nan'

# Apply the function across the DataFrame

mask = train_df.applymap(is_nan_string)

# Filter out rows that contain the string "Nan"

cleaned_df = train_df[~mask.any(axis=1)]

train_df.to_excel('train_4500.xlsx')

eval_df.to_excel('eval.xlsx')

import pandas as pd

df = pd.read_excel('train_df_with_examples_and_intro.xlsx')

df['full_note'] = df['full_note'] + end

train_df.to_excel('dev_df_with_intro.xlsx')

df = pd.read_excel('L3_DPO_dev.xlsx')

df = pd.read_excel('L2_M2_test_Summary_GPT_file.xlsx')

eval_prompt1 = """

Given the following full clinical note, rate the summary from a scale of 1 (the best) to 5 (the worst). The summary should be concise (2-3 sentences) and contain all the information that would be important to an internal medicine physician.

---------

"""

### THIS PROMPT WAS USED #####

example_summary4 = """

Example Summary 1:

This is a 74 year old man with a past medical history of hypertension, chronic obstructive pulmonary disease, chronic kidney disease, coronary artery disease, and abdominal aortic aneurysm status post endovascular repair 8 years ago, admitted to the ICU for decompensated heart failure with pulmonary edema secondary to severe mitral regurgitation, treated with mitral valve repair. During the hospitalization a TEE was performed and complicated by hypopharyngeal injury without esophageal damage, resulting in a retropharyngeal abscess formation that responded to antibiotics and needle aspiration, the patient is currently tolerating a diet of small portions of soft food.

Rating: 1

Example Summary 2:

A 74 year old man comes in with pulmonary edema from heart failure treated with a procedure. He has a history of hypertension. His hospitalization is complicated by a hypopharyngeal injury but is now able to have a soft diet.

Rating: 5

----------

"""

df['tsavage68/Summary4500_M2_200steps_1e7rate_SFT_evaluation'] = 'Nan'

df['mistralai/Mistral-7B-Instruct-v0.2_evaluation'] = 'Nan'

df['tsavage68/Summary4500_M2_1000steps_1e7rate_03beta_CSFTDPO_evaluation'] = 'Nan'

df['meta-llama/Meta-Llama-3-8B-Instruct_evaluation'] = 'Nan'

df['tsavage68/Summary4500_L3_100steps_1e6rate_SFT_evaluation'] = 'Nan'

df['tsavage68/Summary4500_L3_1000steps_1e6rate_01beta_CSFTDPO_evaluation'] = 'Nan'

# Prompt A

for i in range(len(df)):

print(i)

#########################################

completion = client.chat.completions.create(

model="gpt-4o",

messages=[

{"role": "system", "content": "You are an expert physician."},

{"role": "user", "content": eval_prompt1 +example_note+ example_summary4 + df['tsavage68/Summary4500_M2_200steps_1e7rate_SFT'][i]}

],

logprobs=True,

#top_logprobs=1,

seed=1,

temperature = 0

)

df['tsavage68/Summary4500_M2_200steps_1e7rate_SFT_evaluation'][i] = completion.choices[0].message.content

#############

completion = client.chat.completions.create(

model="gpt-4o",

messages=[

{"role": "system", "content": "You are an expert physician."},

{"role": "user", "content": eval_prompt1 +example_note+ example_summary4 + df['mistralai/Mistral-7B-Instruct-v0.2'][i]}

],

logprobs=True,

#top_logprobs=1,

seed=1,

temperature = 0

)

df['mistralai/Mistral-7B-Instruct-v0.2_evaluation'][i] = completion.choices[0].message.content

#############

completion = client.chat.completions.create(

model="gpt-4o",

messages=[

{"role": "system", "content": "You are an expert physician."},

{"role": "user", "content": eval_prompt1 +example_note+ example_summary4 + df['tsavage68/Summary4500_M2_1000steps_1e7rate_03beta_CSFTDPO'][i]}

],

logprobs=True,

#top_logprobs=1,

seed=1,

temperature = 0

)

df['tsavage68/Summary4500_M2_1000steps_1e7rate_03beta_CSFTDPO_evaluation'][i] = completion.choices[0].message.content

#############

completion = client.chat.completions.create(

model="gpt-4o",

messages=[

{"role": "system", "content": "You are an expert physician."},

{"role": "user", "content": eval_prompt1 +example_note+ example_summary4 + df['meta-llama/Meta-Llama-3-8B-Instruct'][i]}

],

logprobs=True,

#top_logprobs=1,

seed=1,

temperature = 0

)

df['meta-llama/Meta-Llama-3-8B-Instruct_evaluation'][i] = completion.choices[0].message.content

#############

completion = client.chat.completions.create(

model="gpt-4o",

messages=[

{"role": "system", "content": "You are an expert physician."},

{"role": "user", "content": eval_prompt1 +example_note+ example_summary4 + df['tsavage68/Summary4500_L3_100steps_1e6rate_SFT'][i]}

],

logprobs=True,

#top_logprobs=1,

seed=1,

temperature = 0

)

df['tsavage68/Summary4500_L3_100steps_1e6rate_SFT_evaluation'][i] = completion.choices[0].message.content

#############

completion = client.chat.completions.create(

model="gpt-4o",

messages=[

{"role": "system", "content": "You are an expert physician."},

{"role": "user", "content": eval_prompt1 +example_note+ example_summary4 + df['tsavage68/Summary4500_L3_1000steps_1e6rate_01beta_CSFTDPO'][i]}

],

logprobs=True,

#top_logprobs=1,

seed=1,

temperature = 0

)

df['tsavage68/Summary4500_L3_1000steps_1e6rate_01beta_CSFTDPO_evaluation'][i] = completion.choices[0].message.content

#############

df.to_excel('/content/drive/MyDrive/SFTvDPO/Summary4500_test_grading.xlsx')

df.to_excel('L3_M2_Summary4500_Test_graded_w_example4.xlsx')
