# Supplementary material for: Fine-Tuning Methods for Large Language Models in Clinical Medicine by Supervised Fine-Tuning and Direct Preference Optimization: Comparative Evaluation
Source: J Med Internet Res. 2025 Sep 23;27:e76048. doi: 10.2196/76048 (PMC12457693; doi:10.2196/76048)
Supplement: Multimedia Appendix 7 [file jmir-v27-e76048-s007.docx]

Please review each of the following patient messages with respect to two the two categories below. If you feel it is impossible to make a determination with the information provided, you can skip the message and mark the “Inappropriate for Triage” column with an “x”.

With what urgency should the message be responded. The two categories are: Urgent (<24 hours) and Non-Urgent (48 – 72 hours). Indications for the urgent group are below

**Indication for Urgent Category (<24 hours)**

- Acute symptoms (ex acute COVD, acute nausea/vomiting, etc)
- Significant lab or vital sign result question that is non-emergent but important (ex. new mass on imaging, new AKI, new LFT rise, hypertensive emergency)
- Time sensitive question (Ex pre-op next day)

Personnel triage should be the personnel with the minimal training necessary to respond to the message. The two categories are: Medical Assistant or Physician. General guidance for medical assistants is below.

**Indications for MA Review**

- Medication Refill Requests (MA can put the order in for the physician to co-sign)
- Requests for faxing or emailing documents
- Providing numbers for referral services / office phone number
- Adjusting patient appointments for non-medical reason (ex the patient requests a change because of
- Requesting general records
- Coordinating vaccinations
