# Supplementary material for: Fine-Tuning Methods for Large Language Models in Clinical Medicine by Supervised Fine-Tuning and Direct Preference Optimization: Comparative Evaluation
Source: J Med Internet Res. 2025 Sep 23;27:e76048. doi: 10.2196/76048 (PMC12457693; doi:10.2196/76048)
Supplement: Multimedia Appendix 8 [file jmir-v27-e76048-s008.docx]

**Table S1.** Sensitivity and specificity for each base model with and without fine tuning for triaging patient messages appropriate for a physician (versus medical assistant).

|  |  | Sensitivity | Specificity |
| --- | --- | --- | --- |
|  | Base | 30% | 99% |
| Llama3 | SFT | 34% | 99% |
|  | DPO | 80% | 84% |
|  |  |  |  |
|  | Base | 20% | 94% |
| Mistral2 | SFT | 25% | 95% |
|  | DPO | 64% | 89% |

**Table S2.** Sensitivity and specificity for each base model with and without fine tuning for triaging patient messages to be marked urgent (versus nonurgent).

|  |  | Sensitivity | Specificity |
| --- | --- | --- | --- |
|  | Base | 89% | 70% |
| Llama3 | SFT | 76% | 69% |
|  | DPO | 63% | 90% |
|  |  |  |  |
|  | Base | 63% | 85% |
| Mistral2 | SFT | 64% | 83% |
|  | DPO | 76% | 78% |

DPO Code:

import os

import yaml

from typing import Dict, Optional

from dataclasses import dataclass, field

import torch

from trl import DPOTrainer

from dotenv import load_dotenv

from transformers import pipeline

from datasets import load_from_disk

from datasets import Dataset, load_dataset

from transformers import AutoModelForCausalLM, AutoTokenizer, HfArgumentParser, TrainingArguments

load_dotenv()

hf_token = ***

pip install ipywidgets

import ipywidgets

#Only if you want to push the model to the hub

from huggingface_hub import notebook_login

notebook_login()

with open("CONFIG.yaml", "r") as stream:

config = yaml.safe_load(stream)

max_steps = config["max_steps"]

optimizer = config["optimizer"]

eval_batch_size = config["eval_batch_size"]

train_batch_size = config["train_batch_size"]

model_name_or_path= config["model_name_or_path"]

if config.get("output_dir", ""):

output_dir = config["output_dir"]

else:

output_dir = f"{max_steps}_DPO"

def get_dataset(train=True):

if train:

dataset = Dataset.from_csv("train.csv")

else:

dataset = Dataset.from_csv("eval.csv")

original_columns = dataset.column_names

def return_prompt_and_responses(samples):

return {

"prompt": [prompt for prompt in samples["full_note"]],

"chosen": samples["chosen"],

"rejected": samples["rejected"],

}

return dataset.map(

return_prompt_and_responses,

batched=True,

remove_columns=original_columns,

)

# 1. load a pretrained model

model = AutoModelForCausalLM.from_pretrained(

model_name_or_path,

low_cpu_mem_usage=True,

torch_dtype=torch.float16,

cache_dir="/workspace",

token=hf_token

)

model.config.use_cache = False

model_ref = AutoModelForCausalLM.from_pretrained(

model_name_or_path,

low_cpu_mem_usage=True,

torch_dtype=torch.float16,

cache_dir="/workspace",

token=hf_token

)

tokenizer = AutoTokenizer.from_pretrained("tsavage68/***", token=hf_token)

tokenizer.pad_token = tokenizer.eos_token

# 2. Load the Stack-exchange paired dataset

train_dataset = get_dataset(train=True)

# 3. Load evaluation dataset

eval_dataset = get_dataset(train=False)

# 4. initialize training arguments:

training_args = TrainingArguments(

per_device_train_batch_size= train_batch_size,

per_device_eval_batch_size= eval_batch_size,

max_steps=max_steps,

logging_steps=10,

save_steps=1000,

gradient_accumulation_steps=2,

gradient_checkpointing=True,

learning_rate=1e-5,

evaluation_strategy="steps",

eval_steps=50,

output_dir=output_dir,

lr_scheduler_type="cosine",

warmup_steps=100,

optim=optimizer,

bf16=True,

remove_unused_columns=True,

run_name=output_dir,

report_to="none"

)

# 5. initialize the DPO trainer

dpo_trainer = DPOTrainer(

model,

model_ref,

args=training_args,

beta=0.1,

train_dataset=train_dataset,

eval_dataset=eval_dataset,

tokenizer=tokenizer,

max_prompt_length=4096,

max_length=1024,

)

dpo_trainer.save_model(output_dir)

# 6. train

dpo_trainer.train()

dpo_trainer.save_model(output_dir)

# 7. save

output_dir = os.path.join(output_dir, "final_checkpoint")

dpo_trainer.model.save_pretrained(output_dir)

dpo_trainer.push_to_hub()

SFT Code:

import os

import yaml

from typing import Dict, Optional

from dataclasses import dataclass, field

import torch

from trl import SFTTrainer

from dotenv import load_dotenv

from transformers import pipeline

from datasets import load_from_disk

from datasets import Dataset, load_dataset

from transformers import AutoModelForCausalLM, AutoTokenizer, HfArgumentParser, TrainingArguments

load_dotenv()

hf_token = ***

pip install ipywidgets

import ipywidgets

#Only if you want to push the model to the hub

from huggingface_hub import notebook_login

notebook_login()

with open("CONFIG.yaml", "r") as stream:

config = yaml.safe_load(stream)

max_steps = config["max_steps"]

optimizer = config["optimizer"]

eval_batch_size = config["eval_batch_size"]

train_batch_size = config["train_batch_size"]

model_name_or_path= config["model_name_or_path"]

if config.get("output_dir", ""):

output_dir = config["output_dir"]

else:

output_dir = f"{max_steps}SFT"

def get_dataset(train=True):

if train:

dataset = Dataset.from_csv("train_df_with_examples_and_intro.csv")

else:

dataset = Dataset.from_csv("eval_df_with_examples_and_intro.csv")

original_columns = dataset.column_names

def return_prompt_and_responses(samples):

return {

"text": [p + " " + t for p, t in zip(samples["full_note"], samples["chosen"])],

}

return dataset.map(

return_prompt_and_responses,

batched=True,

remove_columns=original_columns,

)

# 1. load a pretrained model

model = AutoModelForCausalLM.from_pretrained(

model_name_or_path,

low_cpu_mem_usage=True,

torch_dtype=torch.float16,

cache_dir="/workspace",

token=hf_token

)

model.config.use_cache = False

tokenizer = AutoTokenizer.from_pretrained(***, token=hf_token)

tokenizer.pad_token = tokenizer.eos_token

# 2. Load the Stack-exchange paired dataset

train_dataset = get_dataset(train=True)

# 3. Load evaluation dataset

eval_dataset = get_dataset(train=False)

# 4. initialize training arguments:

training_args = TrainingArguments(

per_device_train_batch_size= train_batch_size,

per_device_eval_batch_size= eval_batch_size,

max_steps=max_steps,

logging_steps=10,

save_steps=1000,

gradient_accumulation_steps=2,

gradient_checkpointing=True,

learning_rate=1e-5,

evaluation_strategy="steps",

eval_steps=50,

output_dir=output_dir,

lr_scheduler_type="cosine",

warmup_steps=100,

optim=optimizer,

bf16=True,

remove_unused_columns=True,

run_name=output_dir,

report_to="none"

)

# 5. initialize the SFT trainer

sft_trainer = SFTTrainer(

model,

args=training_args,

train_dataset=train_dataset,

eval_dataset=eval_dataset,

dataset_text_field="text",

tokenizer=tokenizer,

max_seq_length=1024,

)

sft_trainer.save_model(output_dir)

# 6. train

sft_trainer.train()

sft_trainer.save_model(output_dir)

# 7. save

output_dir = os.path.join(output_dir, "final_checkpoint")

sft_trainer.model.save_pretrained(output_dir)

sft_trainer.push_to_hub()
